# Navigating the future of artificial intelligence technologies for improving the care of older adults

**DOI:** 10.1093/geroni/igaf092

**Published:** 2025-09-25

**Authors:** Michael Abadir, William Dineen, Daniel Myers, Simone Yu, Phillip Phan

**Affiliations:** College of Computer, Mathematical, and Natural Sciences, University of Maryland, College Park, Maryland, United States; Motorola Mobility, Chicago, Illinois, United States; CGS Administrators, Nashville, Tennessee, United States; Samueli School of Engineering, University of California, Los Angeles, California, United States; Carey Business School, Johns Hopkins University, Baltimore, Maryland, United States; Division of Geriatric Medicine and Gerontology, Johns Hopkins University, Baltimore, Maryland, United States

**Keywords:** Technology over the Aging Life Course, Healthcare innovation, Healthcare policy, 4Ms

## Abstract

The rapid aging of the global population presents complex challenges for health systems, families, and societies. Artificial intelligence (AI) technologies—from predictive analytics and conversational agents to robotic caregivers and remote monitoring—offer scalable solutions to support older adults throughout their life courses. This article examines the evolving landscape of AI-enabled care for aging populations, structured around the geriatric 4Ms: what Matters, Medication, Mentation, and Mobility. We synthesize current evidence on the application of AI in personalized medicine, cognitive support, fall detection, and chronic disease management while exploring the cultural, economic, and policy contexts that influence technology adoption. The market for age-related technology is expanding; however, disparities in access, digital literacy, and algorithmic bias risk exacerbating inequities. We identify persistent gaps in implementation, including the underrepresentation of diverse older adults in training data sets, limited longitudinal studies, and a lack of integration across diagnostic and therapeutic systems. We propose a future research agenda centered on five priorities: (1) establishing life-course AI research agendas, (2) promoting inclusive and participatory development processes, (3) advancing gerontological design principles, (4) expanding AI literacy across the aging services workforce, and (5) developing robust ethical and regulatory infrastructures. The article calls for interdisciplinary collaboration among gerontologists, engineers, policymakers, and ethicists to ensure that AI innovations are safe, equitable, and responsive to the lived experiences of older adults. Ultimately, we argue that AI must be implemented not as isolated tools but as components of comprehensive care ecosystems that support aging in place, reduce caregiver burden, and preserve human dignity.

Translational Significance: Artificial intelligence provides scalable solutions to the increasing challenge of caring for older adults amidst caregiver shortages and rising health complexities. This article highlights promising AI technologies in predictive analytics, remote monitoring, robotics, and conversational agents aligned with the 4Ms (what Matters, Medication, Mentation, and Mobility) of geriatric care. The paper outlines a roadmap for integrating AI into person-centered, culturally responsive aging systems by exploring adoption barriers and policy gaps. These insights can inform ethical, economic, and regulatory frameworks that empower individuals, strengthen care organizations, and enhance societal preparedness for population aging while maintaining autonomy, equity, and quality of life.

By 2050, the population aged 65 and over is projected to more than double, reaching 22% of the global population, representing 1.6 billion people (United Nations Department of Economic and Social Affairs, 2023). The challenges associated with providing care for this demographic are urgent, highlighting the critical need for innovations. AI technologies offer scalable solutions to these challenges by effectively addressing the 4Ms (what Matters, Medications, Mobility, and Mentation) of geriatric care, especially when deployed in resource-constrained environments (Abadir & Chellappa, 2024). AI encompasses a broad spectrum of computational technologies that mimic certain aspects of human intelligence, including learning, reasoning, and decision-making. It can also be integrated into wearable monitors, voice, or image-activated assistants, robotic caregivers, and electronic health records (EHR) as clinical ­decision-support tools. The significance of AI technologies lies in their ability to enhance clinical care and support the daily living of older adults. AI-driven tools can monitor physiological signals to detect early warning signs of disease, assist with medication adherence, provide companionship through virtual agents, and support mobility through intelligent robotics. These applications are especially crucial as the aging population grows and the supply of informal and professional caregivers becomes increasingly strained. Such technologies represent significant market opportunities and, therefore, proliferate for businesses in the healthcare sector due to the size and growth of the older adult population (Agarwal, 2018).

This paper discusses AI-based technologies that address the challenges of providing care for older adults over the life course, emphasizing how these technologies can support the 4Ms in the context of the cultural, political, and market factors that influence adoption. We begin by exploring the current state of science on AI applications in aging and identifying gaps in research and implementation. We conclude with a future-­oriented agenda that centers on algorithmic reliability, ethics, and the value of interdisciplinary collaboration.

## The market for technologies to support older adults

The current $740 billion technology market for older adults is diverse and holds significant growth potential (Agarwal, 2018). Home healthcare services account for approximately 45% of the market, with in-home care solutions projected to reach $1.1 trillion by the end of 2030 (BCC Research, 2024). Other parts of the market include assistive devices, such as mobility aids and monitoring systems, which are growing at an annual rate of 7.9% and forecasted to reach $346.4 billion by 2025 (BCC Research, 2024), as well as telehealth and remote care. Regarding the latter, only 1 in 10 older adults currently has access to telehealth, highlighting a significant market opportunity (Fine, 2025). In this milieu, we further explore four types of technologies due to their growth potential and promise in addressing the 4Ms of geriatric care: predictive analytics, conversational AI and cognitive support, robotic and augmented sensory assistance, and remote proactive care (RPC).

### Predictive analytics

Artificial intelligence has demonstrated promising results in predicting health outcomes relevant to older adults. The precision offered by AI is crucial for aging populations, where individual variability in biological aging processes contributes to differences in disease susceptibility, progression, treatment response, and even vulnerabilities to adverse events (Alowais et al., 2023). Such technologies support individualized medicine, which involves tailoring treatment plans to an individual’s phenotype and genotype. By incorporating a patient’s unique personal information into AI algorithms, clinicians can develop treatments that are tailored to the individual, making them more effective, with fewer side effects, and reducing the costs associated with inappropriate or inadequate treatments (Topol, 2019).

With multimodal data, AI decision support tools elevate personalization by incorporating environmental and social determinants of health (SDH) data into prognostics and treatment recommendations. For example, Yuan et al. (2021) developed a predictive model for 30-day hospital readmission risk among older adults by integrating EHR with neighborhood-­level indices such as the Area Deprivation Index. The inclusion of these SDH variables improved model performance and highlighted disparities in healthcare outcomes related to socioeconomic context factors, particularly relevant for aging-in-place initiatives (Yuan et al., 2021). Padula et al. (2021) integrated EHR with environmental data from the EPA and CDC’s Environmental Public Health Tracking Network, including particulate matter exposure and proximity to green spaces. The resulting models were able to identify disparities and predict cardiovascular events in older adults based on their residential location. Hence, while traditional EHRs may not natively contain SDOH or environmental data, machine learning approaches are increasingly capable of merging disparate data sources to provide more holistic, socially aware clinical insights.

Artificial intelligence is especially powerful when working with older adults facing multiple complex morbidities involving contraindicated interventions. The reason is that AI is cognitively agnostic and can detect unanticipated patterns in data to predict better treatment outcomes (Alowais et al., 2023). Machine learning applied to EHRs can identify early indicators of falls, cognitive decline, and unplanned hospitalizations (Topol, 2019). Tools like DeepSurv and random forest classifiers have demonstrated utility in risk stratification, although few have been validated in older, diverse populations.

More recently, generative artificial intelligence (gAI) based on large language models (LLMs) represents a promising frontier for the care of older adults. Rapid advancements in its reliability and accuracy have exceeded experts’ expectations (Mackenzie, 2023). LLMs support clinical decision-making and predictive analytics by ingesting and analyzing vast amounts of multimodal data, including EHR, clinical notes, location, environment, wearable sensors, and SDH, in real-time (Wang et al., 2023). For example, Med-PaLM 2, developed by Google DeepMind, has been tested with synthetic and ­de-­identified real-time EHR data to answer complex medical questions, summarize patient records, and assist in triage. By embedding the LLM into clinical workflows, the model can dynamically access up-to-date patient data from EHRs and generate tailored responses in real time (Singhal et al., 2023). In another example, Nuance’s Dragon Ambient eXperience (DAX) Copilot uses GPT-4 to listen in on doctor–patient encounters (with consent), transcribe conversations in real time, and automatically generate structured clinical documentation within seconds (Microsoft, 2023).

Real-time LLMs, used correctly, help overcome heuristics and biases, supporting early and accurate diagnosis, monitoring disease progression and prognosis, and creating individualized treatment plans (Lee et al., 2020). For instance, detecting presymptomatic signs of cognitive decline enables healthcare providers to intervene at critical moments, thereby improving quality of life (Barnes et al., 2020).

### Conversational AI and cognitive support

Virtual assistants and chatbots are being explored for mental health screening, reducing loneliness, and enhancing cognitive engagement. For example, tools like ElliQ and voice-based AI interfaces have been pilot-tested among isolated seniors with some success, although long-term efficacy data remains limited (Broadbent et al., 2020). More generally, voice-activated virtual home assistants (VHAs), such as Amazon’s Alexa and Google Assistant, embodied in such hardware as Amazon Echo and Google Nest, are widespread, providing older adults with an accessible way to engage with technology. They address daily tasks and help reduce social isolation and loneliness (Corbett et al., 2021). VHAs enable users to perform various tasks, including playing music, setting reminders, and making video calls, which can foster a sense of companionship and connection. These devices benefit cognitive engagement for older adults, particularly those with mobility or cognitive limitations. Research by Pradhan et al. (2020) found that older adults frequently utilized voice assistants for health-­related queries despite facing challenges with remembering commands and experiencing voice recognition errors. Additionally, LLMs can leverage natural language processing and speech capabilities to potentially delay cognitive decline in older adults facing Alzheimer’s disease and related dementias (ADRD) (Singhal et al., 2023). LLMs can also assist with daily living functions, such as reminding individuals to take medications, stick to health routines, and manage daily activities to alleviate caregiver burden (Ayers et al., 2023). Finally, in addition to social engagement and cognitive support, smart home platforms integrated with other devices, such as locks, thermostats, plugs, appliances, blinds, robotic appliances, cameras, and lights, work together to promote safety and support the daily tasks of older adults with various functional limitations.

### Robotic and augmented sensory assistance

Robotic caregiving devices, such as robotic arms for feeding, socially assistive robots (SARs), and mobility aids, are gaining traction, particularly in East Asian settings, as innovative tools to support aging in place and reduce caregiver burden ­(Broadbent et al., 2020). When paired with augmented sensory assistance, robotic systems equipped with enhanced sensing technologies, such as cameras, microphones, haptic sensors, and AI, can act to support or substitute for human sensory functions, including vision, hearing, and touch. These systems are designed to interpret environmental stimuli and deliver processed sensory information to users in ways that improve their perception, interaction, or safety, particularly among individuals with sensory impairments (Kaur et al., 2020). For example, the Obi Feeding Robot features a robotic arm that assists individuals with upper-body mobility impairments by enabling them to eat independently. Users control the robot via switches, and it autonomously selects and delivers food from a segmented plate (Kairy et al., 2019). Similarly, Toyota, which has redefined itself as a mobility company, created the Toyota Human Support Robot, an autonomous mobile robot equipped with a manipulator arm to open doors, retrieve items, and assist users with mobility and daily tasks. The robot is activated via a voice and tablet interface and features autonomous navigation and object recognition (Yamazaki et al., 2014). Finally, Mabu from Catalia Health is a socially assistive robot that engages users in conversations to promote medication adherence, track mood, and deliver personalized health coaching (Robinson et al., 2014). The ability of AI to ingest multimodal, real-time data, as discussed in earlier examples, is making these robotic assistants contextually aware, thereby increasing their capability to act autonomously and anticipate the needs of older adults at home. These technologies are designed to assist older adults with daily tasks, mobility, and cognitive stimulation; in some cases, they also offer companionship (Sawik et al., 2023). SARs have demonstrated the potential to reduce agitation and mitigate feelings of loneliness and depression among individuals with mild cognitive impairment (MCI) and dementia (Sawik et al., 2023). Focus groups of older adults and caregivers further support their integration into home environments, citing improved autonomy and quality of life (Broadbent et al., 2018).

Robotic technologies are increasingly paired with immersive virtual and augmented reality tools to train and rehabilitate older adults. Immersive VR applications, such as motion-based exergames, have shown benefits in improving balance, gait, and cognitive performance, including memory and attention. When integrated with AI and multimodal data inputs, these technologies offer physical and cognitive enhancement opportunities and promote social interaction and psychological engagement through culturally contextualized experiences.

The frontier in this field is the brain-computer interface (BCI), which offers transformative potential for enhancing autonomy and quality of life in older adults with cognitive and motor impairments. These systems facilitate direct communication between the brain and external devices such as wheelchairs, prosthetics, and smart home technologies via neural signals. Noninvasive electroencephalography-based BCIs appeal to older adults due to their accessibility and minimal medical risk (Belkacem et al., 2020). Research indicates that BCIs can support rehabilitation by improving memory, ­attention, and mobility and restoring control over devices for ­individuals with severe motor impairments (Naddaf & Drew, 2024).

### Remote proactive care

Remote health monitoring has increasingly become central to the early detection and management of age-related chronic diseases, including type 2 diabetes (T2D) and ADRD. Additionally, digital monitoring provides real-time, scalable assessments of cognitive, physical, and metabolic health. Paired with AI technologies, remote health monitoring can facilitate proactive care strategies when integrated into smart home platforms and clinical dashboards. Remote monitoring systems can be categorized into active and passive. Active monitoring requires intentional user interaction, such as completing cognitive assessments via smartphones, while passive monitoring employs sensors or devices to gather ambient data autonomously. Both modalities generate data that can power AI systems to provide insight into user health states. By adding continuous data acquisition devices such as wearables or always-on in-home sensors, rich longitudinal data on health and behavior allow for the use of AI to provide early detection of cognitive or physical impairment (Staffaroni et al., 2021).

Wearable devices, including smartwatches, fitness trackers, and continuous glucose monitors, provide granular, real-time insights into physiological markers such as heart rate, activity levels, sleep patterns, and glucose variability (Zhan et al., 2018). These data have demonstrated the ability to detect early signs of neurodegenerative disorders, including changes in gait and balance linked to Parkinson’s disease and AD (Berron et al., 2022). Furthermore, connections between physical activity, glucose fluctuations, and cognitive performance in older adults with T2D are becoming increasingly evident (Brundel et al., 2014).

In-home sensors further enhance passive data collection. For instance, passive infrared (PIR) motion sensors track movement patterns, time spent in various rooms, and social activity, all linked to cognitive decline (Mackin et al., 2018). PIR sensors have also been used to monitor gait variability, an established marker of physical and cognitive deterioration (Papp et al., 2021). Other sensors, such as smart scales, sleep mats, and ambient ­monitors, collect complementary data on weight changes, sleep quality, and heart rate variability.

Smartphones offer a versatile platform for both passive and active monitoring. Behavioral patterns such as reduced phone usage, erratic typing, and diminished engagement with common apps can signal cognitive impairment (Gold et al., 2018). ­Smartphone-based assessments of motor function, including finger tapping and balance tests, also correlate with established clinical metrics (Bunker et al., 2017). In older adults with T2D, smartphone-based tracking of speech, movement, and activity has shown sensitivity to early signs of cognitive decline, including pause lengths and syntactic complexity in speech (Tang et al., 2022).

The coronavirus disease 2019 (COVID-19) pandemic marked a turning point for telemedicine, leading to rapid adoption among patients who could not visit providers due to social distancing mandates. By combining remote monitoring, AI decision support, and remote consultation, which we call RPC, continuous care is extended to patients aging at home. Emerging evidence now suggests that telemedicine not only increases access but also transforms the delivery of safe care to older adults. It reduces hospital-acquired infections, provides greater access for individuals with mobility limitations or those in rural areas, and enhances cost-efficiency for socioeconomically disadvantaged populations (Doraiswamy et al., 2021). RPC equips clinicians to manage chronic conditions like diabetes and heart disease more effectively by detecting changes early and enabling timely interventions without the need for sporadic clinic visits (Doraiswamy et al., 2021).

An increasingly important application of AI for older adults is fall detection, a pressing concern considering that falls are the second leading cause of accidental injury deaths globally and often occur outside clinical settings (Centers for Disease Control and Prevention, 2024). In the United States, one in four adults aged 65 and older falls each year, totaling 36 million incidents, often resulting in fractures or head injuries and costing over $50 billion annually in healthcare expenditures (Florence et al., 2018). AI-enhanced fall detection systems utilize data from wearable sensors, microphones, gyroscopes, and ambient sensors to distinguish between normal movement and fall events. These systems show high sensitivity and specificity (Usmani et al., 2021). More recently, wearables like the Apple Watch Series 4 and newer models now incorporate integrated fall detection (Apple Support, 2024), while in-home solutions use ­pressure-sensitive floor mats, infrared motion detectors, and vision-based systems such as Microsoft Kinect. Other innovations employ depth sensors and thermal imaging to maintain user privacy while accurately identifying falls.

The next frontier in RPC is fall *prediction*, which assesses environmental and individual risk factors before an event occurs. Despite these technological advancements, real-world implementation continues to lag due to privacy concerns and the social acceptability of pervasive surveillance technologies (Pech et al., 2021). Future efforts must consider technical feasibility and the nonclinical contexts of older adults’ lives that influence use.

## Barriers to AI technology adoption by older adults

Adopting AI technologies for older adults is influenced by a complex interplay of sociocultural, political, economic, and technological factors. This section discusses the cultural and community needs, the political, financial, and market, and access to technology barriers to inclusive innovation and ensuring that AI solutions serve aging populations equitably.

### Cultural and community needs

Cultural context and community structure are critical in shaping older adults’ engagement with emerging technologies such as AI. Across urban, suburban, and rural settings, distinct disparities in access, preferences, and digital literacy significantly affect adoption patterns (Binette & Vasold, 2018; Walsh, 2021). A universal challenge identified in the literature is the need for accessibility—both physical (e.g., broadband infrastructure) and cognitive (e.g., familiarity and trust in digital tools). For instance, in rural communities, where older adults often experience isolation and limited healthcare access, the deployment of AI tools, such as GPT-based systems or remote monitoring, may be hindered by inadequate internet connectivity.

In addition to infrastructural challenges, digital literacy and cultural familiarity with technology remain significant barriers. Although promising to improve healthcare access, technologies like telemedicine often face resistance due to unfamiliarity or discomfort with virtual consultations among older populations. Language and cultural alignment also influence adoption, particularly in ethnically diverse communities, necessitating voice assistants and robotic caregivers to be multilingual and culturally sensitive (Lee et al., 2020).

Privacy and trust issues further complicate adoption. Technologies that involve continuous monitoring—such as fall detection systems and voice-activated assistants—raise concerns about surveillance and data misuse, especially among older adults in minority communities (Wang et al., 2019). Clear communication about data usage and a straightforward explanation of benefits are crucial for addressing these issues. Culturally specific household structures also affect adoption dynamics; for example, intergenerational households, common among Hispanic and Asian populations, create opportunities for shared use of technologies that assist older adults and their family caregivers.

Cultural perceptions of caregiving can create resistance to robot technologies. In cultures that prioritize familial care, robotic caregivers may be seen as depersonalizing or inadequate substitutes for human attention (Wang et al., 2019). Designers must show how these tools enhance rather than replace human caregiving (Coghlan, 2022). Similarly, BCIs, which hold promise for individuals with cognitive or motor impairments, may raise ethical concerns in communities that are less familiar with or skeptical of invasive technologies. Personalized medicine powered by AI, which requires genetic data, also faces barriers to adoption in populations with a historical mistrust of medical institutions (Erdmann et al., 2021).

### Political, economic, and market dynamics

Labor market shortages in elder care further intensify the need for technological solutions. As Tang (2024) reports, 74% of firms in the elder care sector employ fewer than 20 people, which limits their capacity to innovate or scale. With the aging population growing faster than the caregiving workforce, AI-driven systems, such as passive home monitoring, can help reduce hospitalizations and improve quality of life. However, their impact remains limited without regulatory frameworks and reimbursement models to integrate such tools into mainstream care delivery. Political and economic structures strongly influence the market trajectory of AI technologies for older adults. Government programs such as Medicare account for an estimated 80% of older adult care revenues (Finch et al., 2017), creating a policy environment where public funding priorities significantly influence which technologies can scale. Nevertheless, solutions that enable aging in place have yet to demonstrate cost-effectiveness to public payers (Agarwal, 2018).

Regulatory uncertainty also hinders innovation. Repealing the Medicare Coverage of Innovative Technology (MCIT) rule eliminated a streamlined pathway for emerging technologies to secure Medicare reimbursement, increasing the time and costs associated with bringing innovations to market (Federal Register, 2021). Without supportive policies, companies face significant challenges in navigating fragmented regulatory frameworks and demonstrating value to cautious public systems (Wang et al., 2019). In contrast, the temporary expansion of telehealth coverage during the COVID-19 pandemic illustrates how flexible policy can accelerate adoption, enhancing the potential for future regulatory adaptations.

Economic access and affordability remain significant concerns. Despite increasing demand, the assistive devices market is projected to grow at an annual rate of 7.9%. However, only 10% of older adults can afford or access the necessary technologies (Binette & Vasold, 2018). This gap underscores the need for public policies that improve affordability, raise awareness, and promote the equitable distribution of technology.

### Access to digital infrastructure

Artificial intelligence technologies depend on access to digital and communications infrastructure. Disparities in digital access and the required literacy continue to hinder adoption. Low-­income, rural, and underserved populations, those who may benefit the most, often face barriers related to affordability, infrastructure, and digital skills (Wang et al., 2019). A significant segment of the older adult population still lacks access, whether by choice, geography, or economic means, to high-speed internet, smartphones, or wearable technologies (Anderson-Lewis et al., 2018). These disparities are exacerbated in bring-your-own-device models, where variations in device type and quality can affect data reliability.

Global broadband gaps, especially in low- and middle-­income countries, exacerbate these inequities (Doraiswamy et al., 2021). Functional and cognitive limitations also pose usability challenges, and the inability to perform physical exams remotely restricts the clinical scope of telemedicine for geriatric care (Doraiswamy et al., 2021). Promising advancements like satellite-based internet (e.g., Starlink) and mobile health infrastructure may help close access gaps. However, widespread and equitable deployment will necessitate simultaneous investments in digital training, customized device design, and culturally responsive user interfaces.

## Barriers to implementation

As we have discussed, AI technologies can transform caregiving for older adults from reactive to proactive approaches by promoting prevention, facilitating early detection of health issues, and supporting aging in place. This can ultimately enhance the quality of life and lower healthcare costs. However, significant gaps in AI science, design, and implementation for older adults must be addressed.

### Lack of fit between AI models and older populations

Current AI models are often developed using data sets that underrepresent older adults, particularly those who are racially and ethnically diverse, cognitively impaired, or from rural areas. This lack of diversity can introduce algorithmic bias and undermine the safety and efficacy of AI applications (Wang et al., 2019). Furthermore, science remains limited due to a reliance on cross-sectional and pilot studies, which fail to capture how older adults interact with AI technologies over time. This data gap has significant implications for designing devices and tools that support the caregiving of older adults.

Many AI tools are often not designed with older adults in mind. There is a lack of focus on codesign with users, accessibility for those with sensory or cognitive limitations, and transparency in data handling. For example, concerns about privacy and surveillance in smart home sensors and fall detection systems can deter adoption (Pech, 2021). Transparent data governance, including clear communication about data use and the implementation of robust privacy safeguards, is essential for earning user trust.

Ethical concerns also include the risk of overreliance on AI in clinical decision-making, where factual errors or “hallucinations” from generative AI systems could threaten patient safety. Although solutions like retrieval-augmented generation are evolving, human oversight remains crucial (Office for Civil Rights, 2016). Developers must prioritize AI as a tool for enhancing personalized, dignified care rather than replacing human connection.

### Technical limitations

Ongoing technical limitations, such as latency, inadequate haptic feedback, and insufficient immersive realism, hinder the effectiveness of virtual and augmented reality (VR/AR) applications for older adults. Although exergames show promise, their social interaction features remain basic. Similarly, BCIs have the potential to expand neuroadaptive support systems but require improved usability in home settings and further advancements in force detection and depth perception (Herweg et al., 2016). Robotic systems face high development and maintenance costs, infrastructure requirements (e.g., home layout adaptation), and ethical resistance stemming from fears of dehumanization (Vandemeulebroucke et al., 2018). The design and engineering gaps carry significant implications for the workforce’s readiness to adopt such technologies, particularly concerning their integration into clinical workflows.

### Adoption in clinical practice

Clinicians, caregivers, and healthcare managers are often ill-equipped to incorporate AI tools into their workflows. Without adequate training and support, AI may increase workload and disrupt care continuity rather than streamline operations. Educational initiatives and professional development programs must prepare the aging services workforce to use these tools effectively. For example, integrated platforms, such as the Collaborative Aging Research using Technology initiative, provide models for combining data from wearables, smartphones, in-home sensors, and patient-reported outcomes into holistic, scalable systems (Staffaroni et al., 2021). Nevertheless, the broader integration of AI tools into care delivery remains fragmented.

### Policy limitations

Finally, as discussed earlier, the policy environment has not kept pace with technological innovation in older adult care. Key concerns, such as liability, reimbursement, consent, data ownership, and regulatory oversight, remain an evolving landscape. Current frameworks are ill-equipped to tackle the ethical complexities introduced by AI in elder care, including transparency, automation bias, and privacy violations (Vayena et al., 2018). Although government initiatives such as the Advanced Research Projects Agency for Health (ARPA-H) aim to catalyze health technology breakthroughs (Lee, 2021), and bipartisan legislation to enhance U.S. technological competitiveness provides funding pathways (Walsh, 2021), innovators must align their ideas with public health priorities and ethical standards.

## Directions for future research

Despite the growing promise of AI in enhancing older adult caregiving, its deployment remains fragmented. AI tools are often implemented in isolation, separate from broader diagnostic, therapeutic, and monitoring frameworks. To fully realize their potential, these technologies must be integrated into user-centered ecosystems that promote continuous, coordinated, and personalized care for older adults. Bridging the persistent divide between technological innovation and practical deployment requires a deliberate and sustained interdisciplinary approach, incorporating gerontology, biomedical engineering, public health, policy, and ethics expertise.

Future research should focus on integrating AI devices, such as wearables, in-home sensors, and virtual assistants, into cohesive, multimodal platforms. These systems must synthesize data across multiple health domains, enabling holistic monitoring and early intervention while reducing the technology burden on older adults and caregivers. Research also needs to consider the affordability of these innovations. Given the potential of RPC to lower avoidable hospitalizations and institutional care, studies evaluating the economic and return-on-investment implications of their adoption will be essential for guiding public and private sector investment.

As discussed, it is equally important to understand the social and cultural dimensions that affect technology adoption. Designs must be tailored to diverse user populations to ensure equitable access and adoption across racial, ethnic, linguistic, and socioeconomic groups (Corbett et al., 2021). Exploring culturally sensitive approaches to device design and deployment will be essential for reducing disparities in access and engagement.

At the same time, there is an urgent need for policy research to identify supportive regulatory pathways. The short-lived MCIT rule, which sought to expedite coverage for breakthrough devices, exemplifies the potential and challenges of regulatory innovation (Federal Register, 2021). Reexamining and building upon such policies could facilitate faster adoption while upholding rigorous safety and efficacy standards.

As AI continues to evolve in robotics, ambient intelligence, and predictive analytics, it is crucial to address ethical implications. These include concerns about surveillance, autonomy, algorithmic bias, and the replacement of human care. Future research must ethically balance innovation with the irreplaceable value of human presence in elder care. To guide the field forward, we propose the following five key directions for future research:

### Establish life-course AI research agendas

There is an urgent need for longitudinal, life-course research that tracks behavioral, psychosocial, and health outcomes associated with AI use in aging populations. Longitudinal studies are essential to evaluate the long-term effects of AI use among older adults, especially those living with multimorbidity or who are homebound. These studies should assess clinical outcomes, quality of life, emotional well-being, functional independence, and caregiver support. Funders and institutions should prioritize research that captures the dynamic interactions between aging trajectories and technological engagement over time (Topol, 2019).

### Promote inclusive and participatory AI development

Older adults, family caregivers, and frontline healthcare workers must be engaged throughout the AI development lifecycle to ensure relevance. Community-based participatory research and human-centered design approaches can support the development of tools that reflect real-world needs and foster user trust. Thorough research on these methodologies must ensure that the data accurately reflects stakeholder concerns. Inclusive data sets and diverse representation in training algorithms are essential to avoid biases that disproportionately harm marginalized populations (Gebru et al., 2021).

### Advance gerontological design thinking

The physical, cognitive, and emotional diversity of aging must be central to AI design frameworks. Collaborative teams, including gerontologists, user-experience experts, industrial designers, and computer scientists, are essential for creating intuitive, adaptive, and accessible technologies. Design principles should emphasize simplicity, interoperability, and personalization, acknowledging older adults’ heterogeneous capabilities and preferences.

### Expand AI literacy and workforce development

As AI becomes increasingly embedded in healthcare delivery, there is a pressing need for education and training programs for clinicians, nurses, direct care workers, and informal caregivers. Therefore, research on instructional design and the social psychology of learning in digital pedagogies should be prioritized. These programs should develop competencies in interpreting AI-generated outputs, ethical reasoning, and technology troubleshooting. Collaborations between academic institutions, industry, and professional associations can facilitate the creation of credentialing programs and continuing education pathways (Rajkomar et al., 2019).

### Develop ethical and regulatory infrastructure

Policymakers and policy researchers must work with gerontologists, ethicists, and technologists to develop governance frameworks that address the unique vulnerabilities of aging populations. Regulatory models should integrate ethical principles such as autonomy, beneficence, justice, and transparency. Research should investigate how these values can be applied in algorithm design, consent processes, and reimbursement mechanisms. Lessons from existing frameworks, like the EU’s Artificial Intelligence Act and U.S. National Academy of Medicine recommendations, can provide foundational guidance (Floridi et al., 2018; National Academy of Medicine, 2022).

## Conclusion

Integrating AI into healthcare presents a significant opportunity to transform how we care for older adults across various settings. As the global population ages and traditional caregiving resources strain under demand, AI-enabled tools, including predictive analytics, robotic assistance, conversational agents, and remote care platforms, offer scalable, personalized, and potentially cost-saving solutions. However, their promise remains inconsistently realized. This paper has shown that AI technologies are not a cure-all but part of a complex sociotechnical ecosystem requiring thoughtful design, equitable implementation, and strong policy support.

To transition from pilots to widespread adoption, we must prioritize life-course research agendas that address the evolving needs, behaviors, and preferences of older adults interacting with technology over time. The future of AI in aging will rely not only on technical sophistication but also on social trust, regulatory foresight, and cultural competence. Research must bridge the gaps between algorithmic design and real-world diversity, ensuring that tools are accessible, inclusive, and respectful of the values of different communities. Policymakers, clinicians, technologists, and leaders in aging services must collaborate to create ethical, user-centered infrastructures for safe deployment.

Importantly, we must also equip the aging services workforce with the skills to harness AI responsibly. Without education and engagement at all levels of care, even the most advanced tools will fail to deliver impact. The next decade represents a critical inflection point. Bold, interdisciplinary action rooted in gerontological insight and committed to health equity will be essential for steering innovation toward the public good. To turn the promise of AI into a reality for older adults, we must shift our approach from experimentation to sustained, inclusive, and evidence-driven transformation. The time to act through research, design, and policy is now.

## Data Availability

There is no data reported in this study.

## References

[igaf092-B1] Abadir P. , Chellappa R. (2024). Artificial intelligence in geriatrics: Riding the inevitable tide of promise, challenges, and considerations. Journal of Gerontology, Series A: Biological Sciences and Medical Sciences, 79, glad279. 10.1093/gerona/glad279 PMC10826903 38289911

[igaf092-B2] Agarwal M. (2018, September 18). The $740 billion senior care market is ripe for disruption, but full of challenges. *Redpoint Ventures*. https://medium.com/redpoint-ventures/the-740-billion-senior-care-market-is-ripe-for-disruption-but-full-of-challenges-a13e3b53548

[igaf092-B3] Alowais S. A. , Alghamdi S. S., Alsuhebany N., Alqahtani T., Alshaya A. I., Almohareb S. N., Aldairem A., Alrashed M., Bin Saleh K., Badreldin H. A., Al Yami M. S., Al Harbi S., Albekairy A. M. (2023). Revolutionizing healthcare: The role of artificial intelligence in clinical practice. BMC Medical Education, 23, 689. 10.1186/s12909-023-04698-1 37740191 PMC10517477

[igaf092-B4] Anderson-Lewis C. , Darville G., Mercado R. E., Howell S., Di Maggio S. (2018). mHealth technology use and implications in historically underserved and minority populations in the United States: Systematic literature review. JMIR mHealth and uHealth, 6, e128. 10.2196/mhealth.8383 29914860 PMC6028762

[igaf092-B5] Apple Support. (2024, October 23). Use fall detection with Apple Watch. *Apple Support*. https://support.apple.com/en-us/108896

[igaf092-B6] Ayers J. W. , Poliak A., Dredze M., Leas E. C., Zhu Z., Kelley J. B., Faix D. J., Goodman A. M., Longhurst C. A., Hogarth M., Smith D. M. (2023). Comparing physician and artificial intelligence chatbot responses to patient questions posted to a public social media forum. JAMA Internal Medicine, 183, 589–596. 10.1001/jamainternmed.2023.1838 37115527 PMC10148230

[igaf092-B7] Barnes D. E. , Zhou J., Walker R. L., Larson E. B., Lee S. J., Boscardin W. J., Marcum Z. A., Dublin S. (2020). Development and validation of eRADAR: A tool using EHR data to detect unrecognized dementia. Journal of the American Geriatrics Society, 68, 103–111. 10.1111/jgs.16108 31612463 PMC7094818

[igaf092-B8] BCC Research. (2024). Elder care services and assistive devices: Global markets (Report No. HLC062E). BCC Publishing. https://academic.bccresearch.com/market-research/healthcare/elder-care-market-report.html

[igaf092-B9] Belkacem A. N. , Jamil N., Palmer J. A., Ouhbi S., Chen C. (2020). Brain computer interfaces for improving the quality of life of older adults and elderly patients. Frontiers in Neuroscience, 14, 692. 10.3389/fnins.2020.00692 32694979 PMC7339951

[igaf092-B10] Berron D. , Ziegler G., Vieweg P., Billette O., Güsten J., Grande X., Heneka M. T., Schneider A., Teipel S., Jessen F., Wagner M., Düzel E. (2022). Feasibility of digital memory assessments in an unsupervised and remote study setting. Frontiers in Digital Health, 4, 892997. 10.3389/fdgth.2022.892997 35721797 PMC9199443

[igaf092-B11] Binette J. , Vasold K. (2018). Home and community preferences: A national survey of adults age 18-plus. AARP Research. https://www.aarp.org/research/topics/community/info-2018/2018-home-community-preference.html

[igaf092-B12] Broadbent E. , Garrett J., Jepsen N., Li O., Ahn H. S., Robinson H., Peri K. (2020). Using robots at home to support patients with chronic conditions: Pilot randomized controlled trial. Journal of Medical Internet Research, 22, e16794. 10.2196/16794 29439942 PMC5829456

[igaf092-B13] Broadbent E. , Garrett J., Jepsen N., Li Ogilvie V., Ahn H. S., ­Robinson H., MacDonald B. (2018). Using robots at home to support patients with chronic conditions: A qualitative analysis of older adults’ and caregivers’ views. Health Expectations, 21, 491–500. 10.1111/hex.12645

[igaf092-B14] Brundel M. , Kappelle L. J., Biessels G. J. (2014). Brain imaging in type 2 diabetes. European Neuropsychopharmacology, 24, 1967–1971. 10.1016/j.euroneuro.2014.09.010 24726582

[igaf092-B15] Bunker L. , Hshieh T. T., Wong B., Schmitt E. M., Travison T., Yee J., Palihnich K., Metzger E., Fong T. G., Inouye S. K. (2017). The SAGES telephone neuropsychological battery: Correlation with in-person measures. International Journal of Geriatric Psychiatry, 32, 991–999. 10.1002/gps.4542 27507320 PMC5299071

[igaf092-B16] Centers for Disease Control and Prevention. (2024). *Older adult fall prevention: Facts about falls*. https://www.cdc.gov/falls/data-research/facts-stats/index.html

[igaf092-B17] Coghlan S. (2022). Robots and the possibility of humanistic care. International Journal of Social Robotics, 14, 2095–2108. 10.1007/s12369-021-00785-5 34221183 PMC8233602

[igaf092-B18] Corbett C. F. , Wright P. J., Jones K., Parmer M. (2021). Voice-­activated virtual home assistant use and social isolation and loneliness among older adults: Mini review. Frontiers in Public Health, 9, 742012. 10.3389/fpubh.2021.742012 34708017 PMC8542756

[igaf092-B19] Doraiswamy S. , Jithesh A., Mamtani R., Abraham A., Cheema S. (2021). Telehealth use in geriatrics care during the COVID-19 ­pandemic—A scoping review and evidence synthesis. International Journal of Environmental Research and Public Health, 18, 1755. 10.3390/ijerph18041755 33670270 PMC7918552

[igaf092-B20] Erdmann A. , Rehmann-Sutter C., Bozzaro C. (2021). Patients’ and professionals’ views related to ethical issues in precision medicine: A mixed research synthesis. BMC Medical Ethics, 22, 116. 10.1186/s12910-021-00679-6 34465328 PMC8406914

[igaf092-B21] Federal Register. (2021). Medicare program; Medicare Coverage of Innovative Technology (MCIT) and definition of “reasonable and necessary.” *Federal Register*. https://www.federalregister.gov/documents/2021/11/15/2021-24916/medicare-program-medicare-coverage-of-innovative-technology-mcit-and-definition-of-reasonable-and

[igaf092-B22] Finch M. , Griffin K., Pacala J. T. (2017). Reduced healthcare use and apparent savings with passive home monitoring technology: A pilot study. Journal of the American Geriatrics Society, 65, 1301–1305. 10.1111/jgs.14837 28407212

[igaf092-B23] Fine T. (2025). Elderly & disabled services in the US—Market research report (2015–2030 ). IBISWorld. https://www.ibisworld.com/united-states/industry/elderly-disabled-services/1607/

[igaf092-B24] Florence C. S. , Bergen G., Atherly A., Burns E., Stevens J., Drake C. (2018). Medical costs of fatal and nonfatal falls in older adults. Journal of the American Geriatrics Society, 66, 693–698. 10.1111/jgs.15304 29512120 PMC6089380

[igaf092-B25] Floridi L. , Cowls J., Beltrametti M., Chatila R., Chazerand P., Dignum V., Luetge C., Madelin R., Pagallo U., Rossi F., Schafer B., Valcke P., Vayena E. (2018). AI4People—An ethical framework for a good AI society: Opportunities, risks, principles, and recommendations. Minds and Machines, 28, 689–707. 10.1007/s11023-018-9482-5 30930541 PMC6404626

[igaf092-B26] Gebru T. , Morgenstern J., Vecchione B., Vaughan J. W., Wallach H., Daumé H. III, Crawford K. (2021). Datasheets for datasets. Communications of the ACM, 64, 86–92. 10.1145/3458723

[igaf092-B27] Gold M. , Amatniek J., Carrillo M. C., Cedarbaum J. M., Hendrix J. A., Miller B. B., Robillard J. M., Rice J. J., Soares H., Tome M. B., Tarnanas I., Vargas G., Bain L. J., Czaja S. J. (2018). Digital technologies as biomarkers, clinical outcomes assessment, and recruitment tools in Alzheimer’s disease clinical trials. Alzheimer’s & Dementia, 4, 234–242. 10.1016/j.trci.2018.03.006 PMC6021547 29955666

[igaf092-B28] Herweg A. , Gutzeit J., Kleih S., Kübler A. (2016). Wheelchair control by elderly participants in a virtual environment with a brain-computer interface (BCI) and tactile stimulation. Biological Psychology, 121, 117–124. 10.1016/j.biopsycho.2016.10.002 27773679

[igaf092-B29] Kairy D. , Tousignant M., Leclerc N., Côté A. (2019). The use of assistive feeding robots: A scoping review. Disability and ­Rehabilitation: Assistive Technology, 14, 244–252. 10.1080/17483107.2017.1410078

[igaf092-B30] Kaur M. , Tiwari A., Mukherjee A. (2020). Assistive robotics: A review of robotic applications for sensory augmentation and rehabilitation. Disability and Rehabilitation: Assistive Technology, 15, 365–374. 10.1080/17483107.2018.153774530638092

[igaf092-B31] Lee B. Y. (2021, July 6). President Biden proposes ARPA-H, new $6.5 billion health entity to transform how research is done. *Forbes*. https://www.forbes.com/sites/brucelee/2021/07/06/president-biden-proposes-arpa-h-new-65-billion-health-entity-to-transform-how-research-is-done

[igaf092-B32] Lee, J., Yoon, W., Kim, S., Kim, D., Kim, S., So, C. H., & Kang, J. (2020). BioBERT: a pre-trained biomedical language representation model for biomedical text mining. Bioinformatics (Oxford, England), 36, 1234–1240. 10.1093/bioinformatics/btz682 31501885 PMC7703786

[igaf092-B33] Mackenzie D. (2023). Surprising advances in generative artificial intelligence prompt amazement—and worries. Engineering, 25, 9–11.

[igaf092-B34] Mackin R. S. , Insel P. S., Truran D., Finley S., Flenniken D., Nosheny R., Ulbright A., Comacho M., Bickford D., Harel B., Maruff P., Weiner M. W. (2018). Unsupervised online neuropsychological test performance for individuals with mild cognitive impairment and dementia: Results from the Brain Health Registry. Alzheimer’s & Dementia: Diagnosis, Assessment & Disease Monitoring, 10, 573–582. 10.1016/j.dadm.2018.05.002 PMC6215059 30406176

[igaf092-B35] Microsoft. (2023). Introducing DAX copilot: Ambient AI powered by GPT-4. https://news.microsoft.com/2023/03/20/ai-nuance-dax-copilot/

[igaf092-B36] Naddaf M. , Drew L. (2024). Second brain implant by Elon Musk’s Neuralink: Will it fare better than the first? Nature, 632, 481–482. 10.1038/d41586-024-02209-5 39112576

[igaf092-B37] National Academy of Medicine. (2022). Artificial intelligence in health care: The hope, the hype, the promise, the peril. National Academies Press. 39146448

[igaf092-B38] Office for Civil Rights. (2016). Individuals’ right under HIPAA to access their health information 45 CFR § 164.524. U.S. Department of Health and Human Services. https://www.hhs.gov/hipaa/for-professionals/privacy/guidance/access/index.html

[igaf092-B39] Padula W. V. , Adams E. K., Carroll N. (2021). Integrating environmental health data into EHRs for improved cardiovascular disease prediction: A machine learning study. Health Affairs, 40, 595–602. 10.1377/hlthaff.2020.01381

[igaf092-B999] Papp K. V. , Rentz D. M., Maruff P., Sun C. K., Raman R., Donohue M. C., Schembri A., Stark C., Yassa M. A., Wessels A. M., Yaari R., Holdridge K. C., Aisen P. S., & Sperling R. A. (2021). The computerized cognitive composite (C3) in A4, an Alzheimer’s disease secondary prevention trial. Journal of Prevention of Alzheimer’s ­Disease, 8, 59–67. 10.14283/jpad.2020.43 PMC7755110 33336226

[igaf092-B41] Pech M. , Sauzeon H., Yebda T., Benois-Pineau J., Amieva H. (2021). Falls detection and prevention systems in home care for older adults: Myth or reality? JMIR Aging, 4, e29744. 10.2196/29744 34889755 PMC8704100

[igaf092-B42] Pradhan A. , Lazar A., Findlater L. (2020). Use of intelligent voice assistants by older adults with low technology use. ACM Transactions on Computer-Human Interaction, 27, Article 31. 10.1145/3373759

[igaf092-B43] Rajkomar A. , Hardt M., Howell M. D., Corrado G., Chin M. H. (2019). Ensuring fairness in machine learning to advance health equity. Annals of Internal Medicine, 169, 866–872. 10.7326/M18-1990 PMC6594166 30508424

[igaf092-B44] Robinson H. , MacDonald B., Broadbent E. (2014). The role of healthcare robots for older people at home: A review. International Journal of Social Robotics, 6, 575–591. 10.1007/s12369-014-0242-2

[igaf092-B45] Sawik B. , Tobis S., Baum E., Suwalska A., Kropińska S., Stachnik K., Pérez-Bernabeu E., Cildoz M., Agustin A., Wieczorowska-­Tobis K. (2023). Robots for elderly care: Review, multi-criteria optimization model and qualitative case study. Healthcare, 11, 1286. 10.3390/healthcare11091286 37174828 PMC10178192

[igaf092-B46] Singhal K. , Azizi S., Tu T., Mahdavi S. S., Wei J., Chung H. W., Scales N., Tanwani A., Cole-Lewis H., Pfohl S., Payne P., Seneviratne M., Gamble P., Kelly C., Babiker A., Schärli N., Chowdhery A., Mansfield P., Demner-Fushman D., Agüera Y Arcas B., Natarajan V. (2023). Large language models encode clinical knowledge. Nature, 620, 172–180. 10.1038/s41586-023-06291-2 37438534 PMC10396962

[igaf092-B47] Staffaroni A. M. , Taylor J. C., Clark A. L., Heuer H. W., Forsberg L. K., Bahl R., Manoochehri M., Vento S., Bajorek L. P., Ong E., You M., Boeve B. F., Kramer J. H., Rosen H. J., Boxer A. L. (2021). A remote smartphone cognitive testing battery for frontotemporal dementia: Completion rate, reliability, and validity. Alzheimer’s & Dementia, 17, e056136. 10.1002/alz.056136

[igaf092-B48] Tang F. , Chen J., Dodge H. H., Zhou J. (2022). The joint effects of acoustic and linguistic markers for early identification of mild cognitive impairment. Frontiers in Digital Health, 3, 702772. 10.3389/fdgth.2021.702772 35224534 PMC8878676

[igaf092-B49] Tang L. (2024). *Retirement communities in the US—Market research report (2014–2029).* IBISWorld. https://www.ibisworld.com/default.aspx

[igaf092-B50] Topol E. J. (2019). Deep medicine: How artificial intelligence can make healthcare human again. Basic Books.

[igaf092-B51] United Nations Department of Economic and Social Affairs. (2023). *World population ageing 2023 highlights.* https://www.un.org/development/desa/pd/sites/www.un.org.development.desa.pd/files/wpa2023_highlights.pdf

[igaf092-B52] Usmani S. , Saboor A., Haris M., Khan M., Park H. (2021). Latest research trends in fall detection and prevention using machine learning: A systematic review. Sensors, 21, 5134. 10.3390/s21155134 34372371 PMC8347190

[igaf092-B53] Vandemeulebroucke T. , de Casterlé B. D., Gastmans C. (2018). How do older adults experience and perceive socially assistive robots in aged care: A systematic review of qualitative evidence. Aging & ­Mental Health, 22, 149–167. 10.1080/13607863.2016.1262820 28282732

[igaf092-B54] Vayena E. , Blasimme A., Cohen I. G. (2018). Machine learning in medicine: Addressing ethical challenges. PLoS Medicine, 15, e1002689. 10.1371/journal.pmed.1002689 30399149 PMC6219763

[igaf092-B55] Wang S. , Bolling K., Mao W., Reichstadt J., Jeste D., Kim H. C., Nebeker C. (2019). Technology to support aging in place: Older adults’ perspectives. Healthcare (Basel, Switzerland), 7, 60. 10.3390/healthcare7020060 30974780 PMC6627975

[igaf092-B56] Wang F. , Casalino L. P., Khullar D., Lin S. (2023). Large language models in medicine. Nature Medicine, 29, 2422–2432. 10.1038/s41591-023-02536-3

[igaf092-B57] Walsh J. (2021, June 8). Senate passes $250 billion bipartisan bill to boost tech competitiveness with China. *Forbes*. https://www.forbes.com/sites/joewalsh/2021/06/08/senate-passes-bipartisan-bill-to-boost-tech-competitiveness-with-china/

[igaf092-B58] Yamazaki K. , Okada K., Inaba M. (2014). Remote home robot services through the combination of the human support robot and a smart device. Proceedings of the IEEE, 102, 1900–1910. 10.1109/JPROC.2014.2361752

[igaf092-B59] Yuan C. , Drake C., Chin M. H. (2021). Incorporating neighborhood-level social determinants of health in predicting hospital readmissions: A machine learning approach. Journal of General Internal Medicine, 36, 482–490. 10.1007/s11606-020-06134-2

[igaf092-B60] Zhan A. , Mohan S., Tarolli C., Schneider R. B., Adams J. L., Sharma S., Elson M. J., Spear K. L., Glidden A. M., Little M. A., Terzis A., Dorsey E. R., & Saria S. (2018). Using smartphones and machine learning to quantify Parkinson disease severity: The mobile Parkinson disease score. JAMA Neurology, 75, 876–880. 10.1001/jamaneurol.2018.0809 29582075 PMC5885192

